# Klotho protects INS-1 pancreatic β-cells from senescence and enhances mitochondrial function

**DOI:** 10.3389/fragi.2025.1512322

**Published:** 2025-02-13

**Authors:** Zhihong Wang, Yunzhi Ni, Yan-Ru Lou, Gerald J. Prud’homme, Qinghua Wang

**Affiliations:** ^1^ Department of Endocrinology and Metabolism, Huashan Hospital, Fudan University, Shanghai, China; ^2^ Department of Clinical Pharmacy and Drug Administration, School of Pharmacy, Fudan University, Shanghai, China; ^3^ Department of Laboratory Medicine and Pathobiology, Keenan Research Centre for Biomedical Science of St. Michael’s Hospital, Toronto, ON, Canada

**Keywords:** cell senescence, Klotho, mitochondria, β-cell, aging

## Abstract

Aging is an important contributing factor for β-cell failure which could lead to the development of type 2 diabetes (T2D). Aging β-cell exhibits signs of senescence and develops senescence-associated secretory phenotype (SASP), causing the senescence and dysfunction of neighboring cells through paracrine action. *Klotho* is recognized as an anti-aging gene, and the corresponding protein is α-Klotho (KL). KL exerts potent anti-aging effects on multiple cell types, but its role in β-cell aging remains unclear. Here we showed that pancreatic INS-1 cell (a rat insulinoma cell line commonly used to study pancreatic β-cell function) developed the typical hallmarks of senescent cells when treated with doxorubicin *in vitro*, and this was accompanied by downregulation of endogenous KL expression. Supplementation with exogenous KL protein protected pancreatic INS-1 cell against senescence, as indicated by downregulation of senescent markers and SA-β-gal staining. Notably, these effects were associated with improved mitochondrial ATP production and mitochondrial dynamic balance, as well as reduced ROS production. Our study further revealed that INS-1 cell treated with doxorubicin exhibited a reduced insulin secretion response to glucose stimulation, while supplementation with KL could reverse this effect. Our results indicate the important role of KL in regulating β-cell senescence and provide new mechanistic insights into its role in β-cell aging.

## Highlights

• Doxorubicin induced senescence in pancreatic INS-1 cell.
• Klotho reduced doxorubicin-induced cellular senescence.
• Klotho promoted glucose stimulated insulin secretion of senescent β-cell.
• Klotho enhanced mitochondrial function of senescent β-cell.

## 1 Introduction

Aging is a major risk factor for type 2 diabetes (T2D) (Aguayo-Mazzucato, 2020). β-cells can compensate for increased metabolic demands by increasing insulin secretion (Wen et al., 2017). However, this compensation is limited by β-cell aging. Cell senescence is a fundamental aging mechanism. During aging, senescent β-cells accumulate and lead to a proliferative response deficiency, which contributes to β-cell dysfunction and impaired glucose homeostasis (Aguayo-Mazzucato et al., 2019).

Cell senescence is a response to different stimuli and cellular stresses, such as DNA damage, oxidative stress, mitochondrial dysfunction or oncogenic activation (Birch and Gil, 2020). Senescent cells have an irreversible cell cycle arrest and lose the ability to proliferate. Although senescent cells cannot divide, they are metabolically active (Kumari and Jat, 2021). Senescent cells can secrete the senescence-associated secretory phenotype (SASP), which includes soluble and insoluble factors (inflammatory cytokines, chemokines and extracellular matrix) that induce dysfunction and senescence of surrounding cells. The markers of senescent cells are not consistent in every senescent tissue. While p16Ink4a and p21Cis1 are known common markers for senescent cells, IGF-1R is a recently described beta cell-specific senescent marker (Aguayo-Mazzucato et al., 2017; Iwasaki et al., 2023).

Senescent β-cells exhibit downregulation of key hallmark β-cell identity genes, including *Insulin 1, Mafa, Nkx6.1*, and *Pdx1*, mainly due to the effects of cellular senescence. Also, senescent β-cells exhibits higher expression of SASP factors, such as TNF-α, IL-6, CXCL1 (Aguayo-Mazzucato et al., 2019; Thompson et al., 2019). Through SASP, these cells may promote chronic, low-grade sterile inflammation. Importantly, senescent β-cell have impaired function, characterized by a lower insulin secretion response to glucose (Aguayo-Mazzucato et al., 2019). Therefore, senescent β-cells may link aging, inflammation, and depressed β-cell function as seen in T2D. However, the mechanism of β-cell senescence remains largely unknown, and there currently no effective intervention for aging-associated β-cell failure.

*Klotho* is an anti-aging gene that encodes a single-pass transmembrane protein (Kuro-o 2010; Cheikhi et al., 2019). The corresponding protein α-Klotho (KL) is expressed in the kidneys, brain, pancreas and several other tissues (Lim et al., 2015). KL has two different forms, the transmembrane form and the soluble form (s-KL). The transmembrane single-pass protein is 130 kDa and consists of 1,012 amino acids, with a short cytoplasmic segment and a long extracellular component containing two domains, KL1 and KL2 (Chen et al., 2018). The extracellular portion can be cleaved by ADAM10 and ADAM17 proteases and release the soluble form of KL (s-Klotho) (Dalton et al., 2017). s-KL in the circulation can play multiple roles as an endocrine hormone. In humans, the KL level declines with age. Homozygous hypomorphic *klotho* mutant mice show premature aging. In human T2D and db/db T2D mice, β-cell-specific expression of KL is decreased. Forced overexpression of KL improved β-cell function in db/db mice (Lin and Sun, 2015).

In this study, we investigated whether KL could play a role in β-cell senescence and explored the mechanisms. We used doxorubicin to induce cell senescence. Doxorubicin is an antibiotic chemotherapeutic agent that induces cellular senescence via inhibition of topoisomerase II and induction of DNA damage (Ghosh et al., 2016; Aguayo-Mazzucato et al., 2019). Mitochondria have a critical role in cellular senescence due to excessive reactive oxygen species (ROS) generation from oxidative phosphorylation. Conversely, senescent cells themselves generate increased ROS levels, primarily due to mitochondrial dysfunction. The elevated ROS generation can activate key signaling pathways regulating senescence, such as P16, P53 and P21 (Macip et al., 2002; Macip et al., 2003; Takahashi et al., 2006). We also examined whether KL could regulate mitochondrial dynamics and ROS production. Our results suggest that KL has a protective effect on doxorubicin-induced cellular senescence, as shown by increased glucose-stimulated insulin secretion, which may be associated with improved mitochondrial function and reduced ROS production. Therefore, KL could be used as a promising strategy for protecting β-cell from stress-induced senescence, and aging associated β-cell failure.

## 2 Materials and methods

### 2.1 Cell culture

INS-1 cells were cultured in RPMI 1640 medium supplemented with 10% fetal bovine serum (FBS), 11.1 mM D-glucose, 100 U/mL penicillin, 100 μg/mL streptomycin, 10 mM HEPES, 2 mM L-glutamine, 1 mM sodium pyruvate and 50 µM 2-mercaptoethanol (All from ThermoFisher) as described previously (Wang et al., 2023). For Klotho-treated culture, INS-1 cells were cultured in RPMI 1640 medium supplemented with 10% FBS and subjected to Klotho (1 μM/2 μM, 5334-KL-025, RD systems) treatment in the absence or presence of 30 nM doxorubicin (MCE, MedChemExpress) for 16 h. The soluble recombinant protein (s-KL) we used consists of both KL1 and KL2 domains.

### 2.2 SA-β-gal assay

Cells were seeded in 24-well culture plates and treated with 1 μM or 2 μM kL in the presence of 30 nM doxorubicin. Cells were washed with PBS and fixed in SA-β-galactosidase (SA-β-gal) staining solution for 15 min at room temperature. Then, the cells were washed three times with PBS and incubated with SA-β-gal staining solution (Senescence β-Galactosidase Staining Kit, Cell Signaling Technology) for 16–20 h at 37°C. For 24-well plates, 500 µL SA-β-gal staining solution was added to each well. After overnight incubation, the cells were washed with PBS and observed under a bright field microscope.

### 2.3 Immunofluorescence staining

For INS-1 cell immunofluorescence staining, INS-1 cells were plated onto cover slides. After treatment with doxorubicin in the absence or presence of KL, INS-1 cells were washed with PBS, fixed and permeabilized with 4% paraformaldehyde for 15 min and gently washed with PBS. Cells were blocked with blocking solution (10% normal goat serum, 0.1% Triton X-100 in PBS) for 1 h at room temperature, and rabbit anti-Ki67 (1:100, Thermo Fisher), rabbit anti-P53 (1:100, Cell Signaling Technology), rabbit anti-P21 (1:100, Cell Signaling Technology), rabbit anti-γ-H2AX (1:100, Cell Signaling Technology) and rabbit anti-Klotho (1:200; Cell Signaling Technology) were added overnight at 4°C. The cells were washed three times with PBS for 5 min, followed by Goat Anti-Rabbit IgG H&L (Alexa Fluor^®^ 488 (1:1000; ab150077, Abcam) and DAPI (1 μg/mL, 4083S, Cell Signaling Technology). All immunofluorescent images were captured by an Olympus upright BX50 fluorescence microscope (Olympus, Huashan Hospital, Shanghai, China) at ×40 magnification.

### 2.4 qPCR analysis

RNA isolation and reverse transcription from 1 μg RNA was performed using TRI Reagent (Sigma Aldrich) and reverse transcriptase (Invitrogen, MA, United States), respectively, according to the manufacturer’s instructions. Real-time qPCR reaction was conducted on (Applied biosystems, 7,500 Real Time PCR System, CA, United States) in 10 μL volume of SYBR green PCR reagents (Thermo scientific, MA, United States) under conditions recommended by the supplier. For IGF-1R, P53, P21, TNF-α and GAPDH mRNA detection, the following primers were used: 5’CGG CAG AGC AAA GGA GAC ATA3’ (IGF-1R forward), 5’TGG TGG AGG TGA AAC GGA 3’ (IGF-1R reverse); 5’ACC AGC ACA AGC TCC TCT CC3’ (P53 forward), 5’AAG GCC CTC ATT CAG CTC TCG3’ (P53 reverse); 5’GGC AAG AGT GCC TTG ACG AT3’ (P21 forward), 5’CCT CTT GAC CTG CTG TGT CG3’ (P21 reverse); 5’GAC​GTG​GAA​CTG​GCA​GAA​GAG3’ (TNF-α forward), 5’ TTG​GTG​GTT​TGT​GAG​TGT​GAG3’ (TNF-α reverse); 5’AAC​TTT​GGC​ATT​GTG​GAA​GG3’ (GAPDH forward) and 5’ACA​CAT​TGG​GGG​TAG​GAA​CA3’ (GAPDH reverse). Results were normalized to GAPDH and relative quantification analysis was performed using the 2^−ΔΔCt^ method.

### 2.5 Western blot analysis

Protein was extracted from INS-1 cells using radioimmune precipitation assay lysis buffer (Beyotime Biotechnology., Ltd.) supplemented with protease and phosphatase inhibitors (Millipore). The total protein concentration was determined using a Pierce BCA Protein Assay kit (Thermo Scientific). Equal amounts of protein (10 µg) were separated by 12% SDS‒PAGE, transferred onto PVDF membranes, and immunoblotted with rabbit anti-Klotho (1:1000; Cell Signaling Technology), anti-P53 (1:1,000, Cell Signaling Technology), anti-P21 (1:1000, Cell Signaling Technology), anti-IGF-1R (1:1000, Proteintech), anti-MFN2 (1:1000, Cell Signaling Technology), anti-OPA1 (1:1000, Abcam) and anti-p-DRP-1(Ser616) (1:1000, Cell Signaling Technology) antibodies at 4°C overnight. Then, the cells were incubated with horseradish peroxidase-conjugated anti-rabbit (1:5000) and anti-mouse (1:5000; both from Jackson Labs) antibodies for 1 h at room temperature. Immunoreactive signals were detected using enhanced chemiluminescence reagents (EMD Millipore; Merck KGaA). Finally, densitometric analysis of the protein bands was performed using ImageJ (NIH).

### 2.6 Measurement of mitochondrial ROS

Mitochondrial ROS in INS-1 cells was determined by Mito-Sox staining. INS-1 cells were cultured in 24-well plates with different treatments. Then, the cells were incubated with 5 μM Mito-Sox (Invitrogen, M36008) for 20 min at 37°C in the dark. Finally, the sample was randomly photographed, and the fluorescence intensity was analysed from five fields of each group using ImageJ software in three independent experiments.

### 2.7 Detection of cellular ATP levels

ATP levels in INS-1 cell lysates were measured using a luminometer (Synergy H4, BioTek Instruments, Inc., United States) according to the manufacturer’s instructions (S0027, Beyotime Biotechnology). Total protein was extracted from INS-1 samples for normalization before ATP assay. According to the manufacturer’s instructions, after centrifugation to remove cell debris, the supernatant was mixed with the substrate solution. The luminescence of the solution was measured using a luminometer with an integration time of 1 s per well. Protein content was determined using the BCA Protein Assay Kit (P0012S, Beyotime), and the ATP concentration was then converted to nanomoles of ATP per microgram of protein.

### 2.8 Cell viability

Cell viability was assessed by CCK8 assay. For the CCK8 assay, experiments were performed in 96-well plates, and INS-1 cells were serum-starved overnight before treatment with various concentrations of Dox for 24 h. Samples of conditioned media were collected for the measurement of viability (CCK8 assay kit, Beyotime), an indicator of cell viability.

### 2.9 Immunostaining of pancreatic Klotho and IGF-1R

Pancreatic sections (4 μM) were incubated with rabbit anti-Klotho (1:100; Cell Signaling Technology), rabbit anti-IGF-1R (1:200; Proteintech), and mouse anti-insulin (1:1000; Proteintech), followed with Goat Anti-Rabbit IgG H&L (Alexa Fluor^®^ 488) (1:1000; Abcam) and Goat Anti-Mouse IgG H&L (Cy3^®^) (1:1,000; Abcam). All immunofluorescent images were captured by an Olympus upright BX50 fluorescence microscope (Olympus, Huashan Hospital, Shanghai, China) at ×40 magnification.

### 2.10 Glucose-stimulated insulin secretion (GSIS)

Cells were cultured in 12-well cluster dishes and incubated with 1 μM or 2 μM Klotho in the presence of 30 nM doxorubicin for 16 h. Cells were washed three times with HEPES balanced salt solution (HBSS) containing 125 mM NaCl, 5.9 mM KCl, 1.28 mM CaCl_2_, 1.2 mM MgCl_2_, 25 mM HEPES (pH 7.4), 2.8 mM glucose and 0.1% fatty acid free BSA (Boehringer Mannheim, GmbH, Germany). The cells were then preincubated with HBSS for 30 min at 37°C and then stimulated with different concentration of glucose (2.8, 16.7 mM) for 2 h at 37°C. Each condition was performed insulin radioimmunoassay. Insulin radioimmunoassay was conducted according to the kit’s instructions (Crystal Chem Inc.).

### 2.11 Statistical analysis

Data are expressed as the means ± SEMs. P values were determined by two-tailed Student’s t test or one-way ANOVA with Bonferroni posttest for multiple comparisons. P < 0.05 was considered statistically significant.

## 3 Results

### 3.1 Doxorubicin induced senescence-associated hallmarks and decreased Klotho levels in INS-1 cell

**FIGURE 1:**
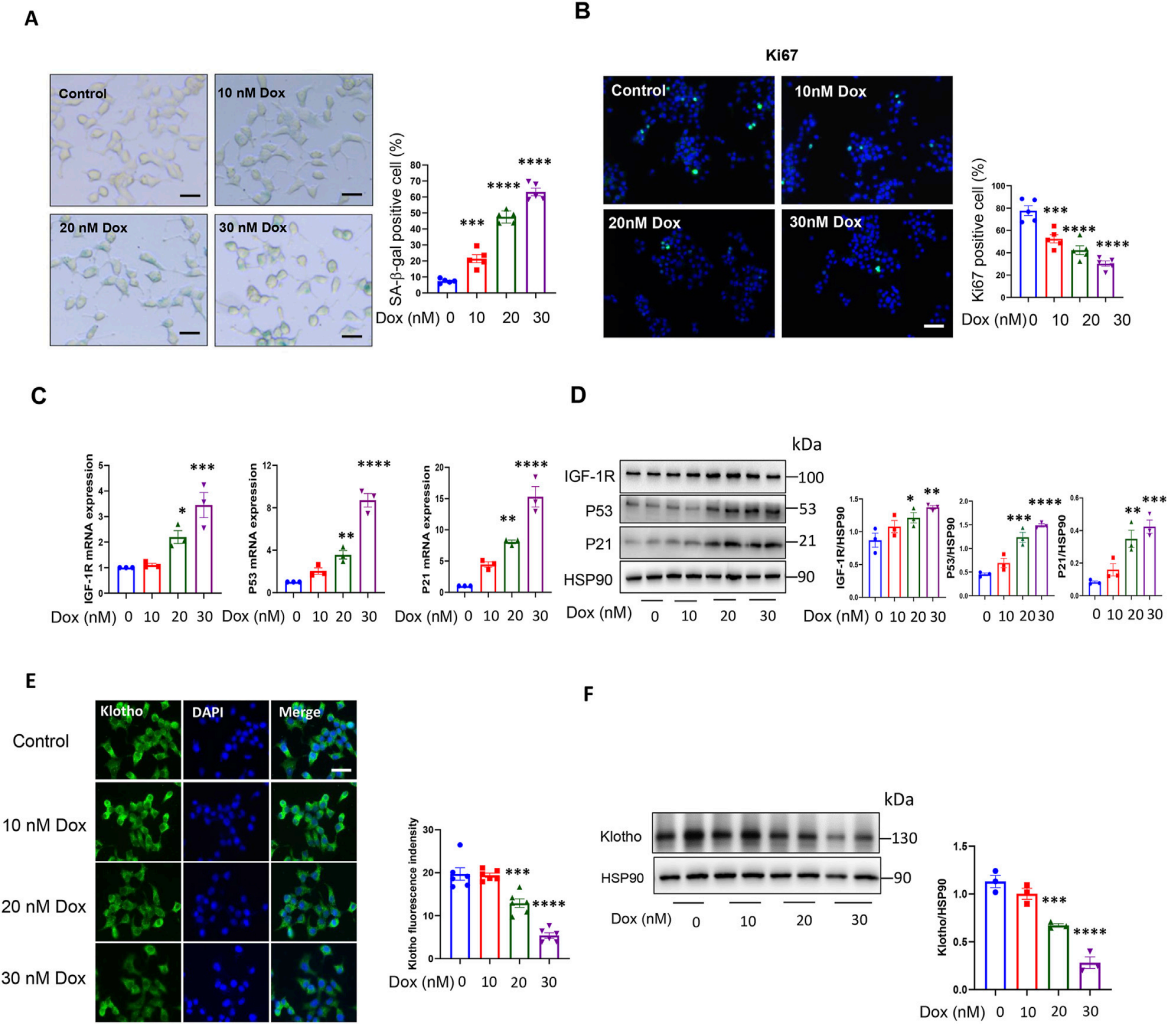
Doxorubicin induced INS-1 cell senescence and decreased KL expression. **(A)** Representative images (left) and quantification (right) of SA-β-gal staining of INS-1 cells after doxorubicin treatment; Scale bar: 12.5 μM; **(B)** IF staining of cell proliferation marker Ki67; Scale bar: 50 μM and **(C)** qPCR analysis of cellular senescence marker: IGF-1R, P53 and P21 after doxorubicin treatment; **(D)** Western blot analysis of cellular senescence marker: IGF-1R, P53 and P21 after doxorubicin treatment; **(E)** IF staining of KL in INS-1 cells after doxorubicin treatment; Scale bar: 25 μM; **(F)** Western blot analysis of KL Data are presented as means ± SEM. **P < 0.01, ***P < 0.001, ****P < 0.0001. Dox, Doxorubicin.

It has been reported that cellular senescence can be induced in β-cell line by doxorubicin (Dox) treatment (Aguayo-Mazzucato et al., 2019). We conducted a dose response experiment with different doses of doxorubicin (Dox) in INS-1 cell to determine the optimal dose of Dox we use in this study. Our results showed that the number of positive SA-β-gal cells was significantly increased after 24 h of Dox treatment (Figure 1A). Since senescent cells lose the ability to proliferate, we also examined the expression of Ki67, a widely used marker for cell proliferation. As shown in Figure 1B, the expression of Ki67 decreased significantly. We also analysed the expression of crucial pathway P53-P21 related to cellular senescence. QPCR and Western blot analysis showed that the expression of P53 and P21 was significantly increased after Dox treatment (Figures 1C, D). These results demonstrated that Dox could induced senescence in INS-1 cell. And 30 nM Dox did not exhibit toxicity to INS-1 cell (Supplementary Figure S1). Overall, the concentration of Dox (30 nM) was chosen for the remaining experiments in this study.

In order to investigate the role of KL in senescent β-cells, we examined the expression of KL in INS-1 cells with Dox treatment. As shown in Figures 1E, F, IF staining and Western blot showed that the expression of KL was decreased significantly. These results indicated that KL may play an important role in process of β-cell senescence.

### 3.2 KL reduced doxorubicin-induced cellular senescence as characterized by the SA-β-gal assay

**FIGURE 2:**
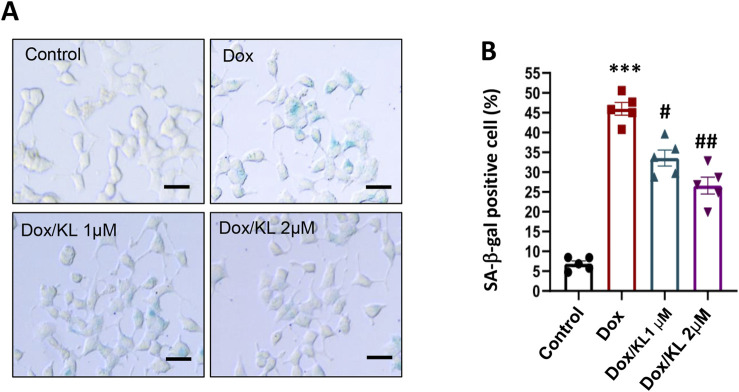
KL attenuates senescence-associated signatures in INS-1 cells after doxorubicin treatment. Representative images **(A)** and quantification **(B)** of SA-β-gal staining of INS-1 cells after KL treatment. Scale bar: 12.5 μΜ. Data are presented as means ± SEM and n = 5 for each of the four groups. ***P < 0.001, #*P* < 0.05, ##*P* < 0.01. KL, Klotho.

To explore the effect of KL on β-cell senescence, cultured INS-1 cells were treated with 30 nM Dox in the presence of 1 µM or 2 µM recombinant human KL protein for 24 h. Then, an SA-β-gal assay was used to detect the number of senescent cells in each group. Very few INS-1 cells were SA-β-gal positive in the non-treatment control group. The number of SA-β-gal-positive cells in the Dox-treated group was significantly increased, while after KL treatment, the number of SA-β-gal-positive cells was much lower than that in the untreated group (Figure 2).

### 3.3 KL reduced doxorubicin-induced cellular senescence and DNA damage

**FIGURE 3:**
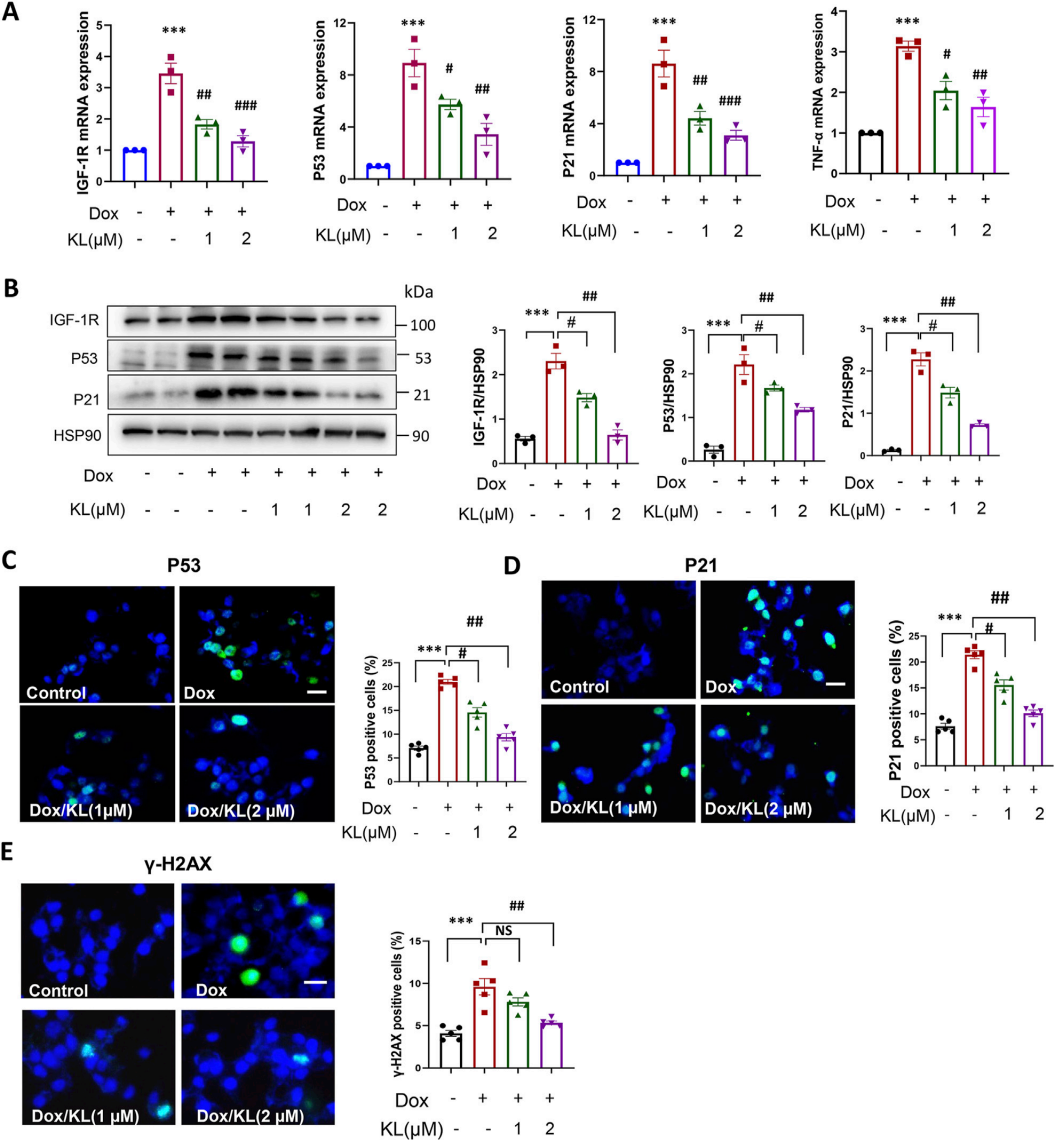
KL reduced doxorubicin-induced cellular senescence characterized by senescence regulators. **(A)** qPCR analysis of cellular senescence marker IGF-1R, P53, P21 and TNF-α in INS-1 cells after doxorubicin and KL treatment; **(B)** Western blot analysis of cellular senescence marker IGF-1R, P53 and P21 in INS-1 cells after doxorubicin and KL treatment; **(C–E)** IF staining of P53, P21and γ-H2AX in INS-1 cells after doxorubicin and KL treatment. Scale bar: 25 μM. Data are presented as means ± SEM, values of three independent experiments are shown. ***P < 0.001, #*P* < 0.05, ##*P* < 0.01, ##*P* < 0.001.

Next, we performed qPCR and Western blotting experiments to confirm the effect of KL on senescent β-cell characterized by senescence markers. qPCR and Western blotting showed that KL significantly reduced the expression of senescent marker IGF-1R, and the expression of pathway P53-P21 and TNF-α compared to the nontreatment (Figures 3A, B). Moreover, IF staining confirmed that KL significantly reduced the numbers of P53- and P21-positive cells (Figures 3C, D). One of the well-known effects of Dox is its ability to induce DNA damage, which serves as a marker for senescent cells. We examined the DNA damage marker γ-H2AX, and the results showed that Dox indeed promoted the expression of the DNA damage marker γ-H2AX, while 2 μM KL could significantly reduce the expression of γ-H2AX (Figure 3E). All of the above proved that Dox promoted the senescence of INS-1 cells *in vitro* and KL could reduce senescence.

### 3.4 KL enhanced mitochondrial function and promoted glucose stimulated insulin secretion

**FIGURE 4:**
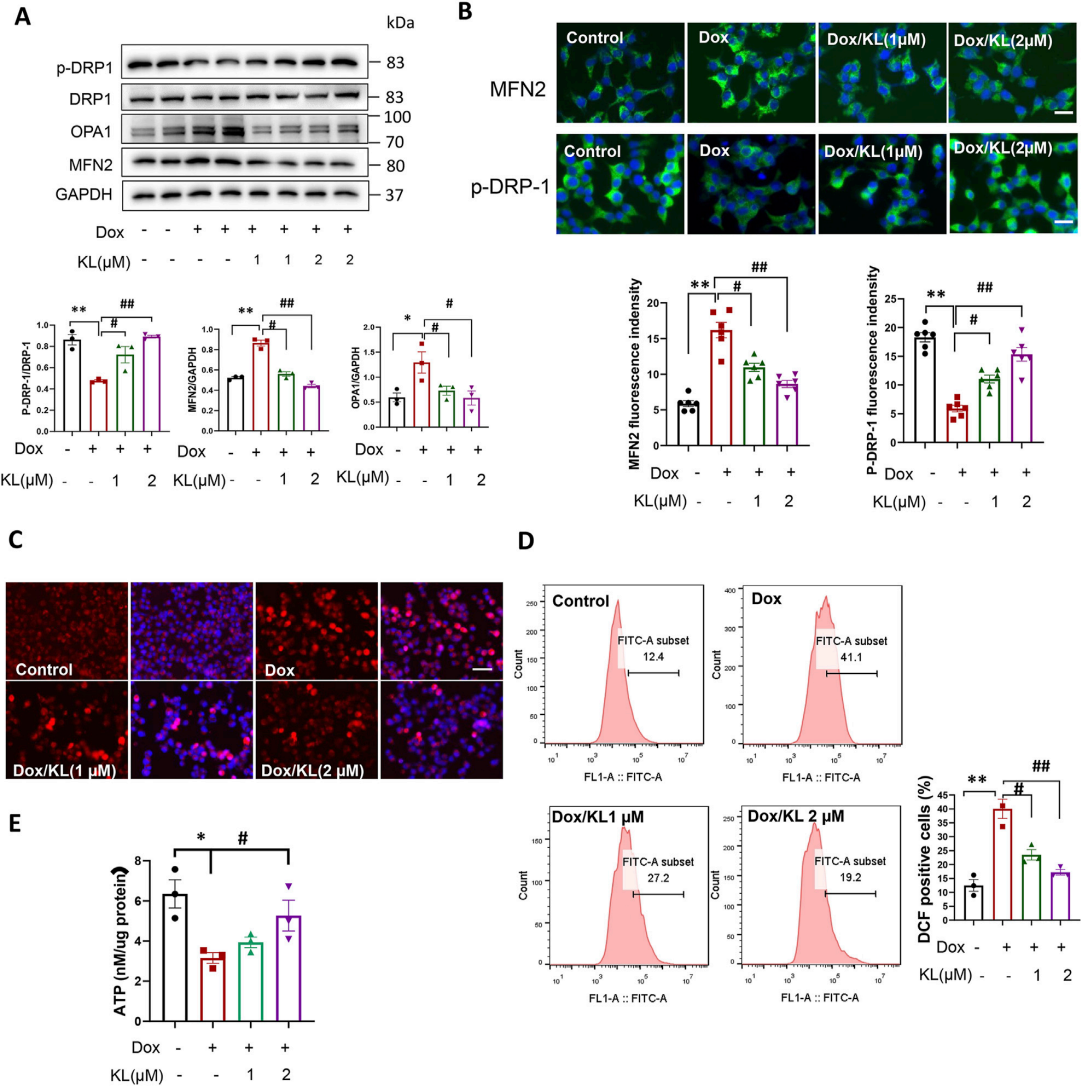
KL regulated mitochondrial dynamics and reduced ROS generation. **(A)** Western blot analysis of mitochondrial fission protein p-DRP-1 and mitochondrial fusion protein MFN2 and OPA1 in INS-1 cells after doxorubicin and KL treatment; **(B)** IF staining of p-DRP-1 and MFN2 in INS-1 cells after doxorubicin and KL treatment, Scale bar: 12.5 μM; **(C)** Mito-Sox staining (DCF) of INS-1 cells after doxorubicin and KL treatment, Scale bar: 50 μM; **(D)** ROS generation was measured by flow cytometry; **(E)** Cellular adenosine triphosphate (ATP) content of INS-1 cells with different treatments. Data are presented as means ± SEM, values of three independent experiments are shown. *P < 0.05, **P < 0.01, #*P* < 0.05, ##*P* < 0.01.

To explore the underlying mechanism by which KL reduced β-cell senescence, mitochondrial dynamics and ROS generation were initially evaluated (Sahu et al., 2018; Lee et al., 2021). As shown in Figures 4A, B, Western blot analysis and IF staining showed that compared with the control, the expression of the mitochondrial fusion protein MFN2 was upregulated after Dox treatment for 24 h, suggesting that the occurrence of mitochondria fusion and KL significantly reduced this upregulation. At the same time, we assessed changes of mitochondrial fission. The results showed KL treatment upregulated the expression of the mitochondrial fission proteins OPA1 and p-DRP-1 (ser 616), when DRP1 is phosphorylated at Ser616, its GTPase activity increases, promoting mitochondrial fission.

We used Mito-Sox to detect ROS production in INS-1 cells. As shown in Figure 4C, the ROS level was significantly enhanced in cells treated with Dox, suggesting that ROS may be involved in the regulation of INS-1 cell senescence. After KL treatment, ROS production was reduced significantly. Additionally, we used flow cytometry to detect ROS production. As shown in Figure 4D, ROS production was significantly increased in cells treated with Dox, and KL reduced ROS production. We also detected the ATP levels in senescent cells. Our date showed that Dox could reduce ATP content significantly, while KL elevated ATP content in cells treated with Dox (Figure 4E).

**FIGURE 5:**
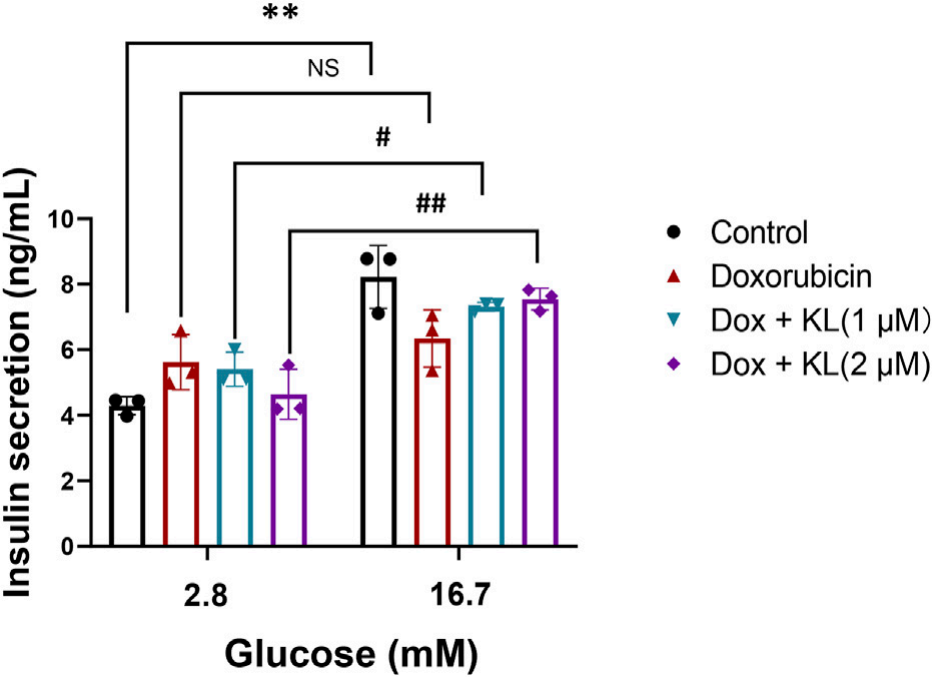
KL increased glucose stimulated insulin secretion in INS-1 cells treated with Dox. GSIS was measured in the absence or presence of KL in INS-1 cells treated with Dox. Data are presented as means ± SEM, values of three independent experiments are shown. **P < 0.01, #*P* < 0.05, ##*P* < 0.01.

We further explored whether KL could regulate insulin secretion in cultured β-cells, we performed GSIS in INS-1 cells treated with Dox and KL. We confirmed that Dox treatment reduced insulin secretion significantly, while insulin secretion increased after KL treatment (Figure 5). The results demonstrated that KL promoted insulin secretion.

## 4 Discussion

We have previously reported that s-KL was effective in the treatment of NOD mice through increasing β-cell mass and reducing islet inflammation (Prud’homme et al., 2020). Other studies have shown that overexpression of KL in pancreatic β-cells could decrease hyperglycemia and enhance glucose tolerance in *db/db* mice (Lin and Sun, 2015). *In vitro*, KL promoted β-cell proliferation, decreased β-cell apoptosis, and enhanced glucose-stimulated insulin secretion (Lin and Sun, 2012; Lin and Sun, 2015). However, KL’s role in β-cell senescence is largely unknown. In this study, we found that supplement with exogenous KL reduced INS-1 cell senescence, indicated by SA-β-gal assay and down expression of P21, P53 and IGF-1R. And these effects were associated with the improvement of mitochondrial function.

In mice, KL deficiency is characterized by accelerated aging, while KL overexpression increases lifespan and delays onset of many age-related diseases (Kurosu et al., 2005). KL also can attenuate or alleviate deleterious changes with aging and disease (Lim et al., 2012; Sahu et al., 2018). In this study, we found that the expression of KL was significantly decreased in β-cells of aged mice. Importantly, along with reduction of KL, senescent β-cells accumulate in aged mice (Supplementary Figure S2). However, the mechanism underlying β-cell aging remains unclear. Mitochondria may be an important mechanism for senescent β-cells since our data showed there were mitochondrial dysfunctions in Dox treated INS-1 cells, including decreased ATP production, increased ROS generation. Importantly, Dox treatment caused mitochondrial dynamic disorder, leading to mitochondrial structural abnormalities, ultimately resulting in oxidative stress and mitochondrial dysfunction.

Mitochondrial dysfunction is a hallmark of aged tissue phenotype, and involve in cell aging process. Mitochondria are the key organelles to produce and supply adenosine triphosphate (ATP). In β-cells, glucose is taken up through GLUT1 (GLUT2 in rodent) on the cell membrane, then, participates in citric acid cycle and causes a rapid increased ATP production, which promotes plasma membrane depolarization and calcium influx triggering insulin exocytosis (Zhu et al., 2021). Defects in mitochondrial function impair this metabolic coupling, and insufficient insulin fails to compensate the demand for insulin, ultimately hyperglycemia occurs (Kalwat and Cobb, 2017; Zhu et al., 2021). In this study, mitochondrial ATP production of senescent β-cell induced by Dox was significantly decreased. And KL could reverse this effect. A previous study has reported that KL could preserve mitochondrial function and maintain the normal structure of mitochondrial, thus protecting against renal tubular epithelial cell senescence in chronic kidney disease (CKD) (Miao et al., 2021).

The prevalence of T2D increases with age. In the elderly, impairment of β-cell function and reduced β-cell mass contribute to diabetes (Zhu et al., 2021). The contribution of β-cell senescence is getting increasing attention (Imai, 2020). In a recent study, Aguayo-Mazzucato et al. explored the roles of senescent β-cells in T2D (Aguayo-Mazzucato et al., 2019). First, the authors sorted senescent β-cells from 7- to 8-month-old mice based on β-galactosidase (β-gal) activity. The number of β-gal positive cells accounts for 8%–10% of the total β-cells. These β-cells have an upregulation of genes related to aging and senescence. Importantly the SASP protein secretion also significantly increased, which induced dysfunction and senescence in their neighboring cells. Then, the authors administered senolytic therapy by using ABT263 to eliminate senescent β-cells, in 15- to 18- month-old mice (Aguayo-Mazzucato et al., 2019). Glucose-stimulated insulin secretion was augmented and β-cell aging and SASP indices was improved. However, senolytic therapies have a major limitation as they target cells and tissues indiscriminately and broadly, and they target pathways that can be upregulated in all cell types.

Interestingly, new drugs are emerging that are capable of manipulating the process of senescence in the context of aging (Kudlova et al., 2022). These include CDK4 inhibitors (similar in principle to p16 activation) aimed at selective elimination of senescent cells (Baker et al., 2016). Senolytic drugs could not only reduce senescent cell burden, but also increase KL in urine, kidney, and brain of mice (Zhu et al., 2022). Importantly, senolytic drug also increased KL in the urine of patients with idiopathic pulmonary fibrosis (IPF), a disease linked to cellular senescence (Zhu et al., 2022). However, there is no direct evidence supporting the effect of senolytic drugs on increasing KL specifically in β-cells. Future studies need to explore whether senolytic drugs influence KL levels in β-cells, which may provide deeper insights into their potential for improving β-cell function and combating age-related decline in pancreatic function. Notably, KL treatment could improve β-cell function and glucose homeostasis, suggesting it would be effective in metabolic diseases.

While the *in vitro* data presented in our study provide valuable insights into the potential role of Klotho in protecting β-cells from senescence, several important considerations must be acknowledged. One limitation is that our findings were derived from immortalized β-cell lines. Immortalized cell lines are useful for initial screenings and mechanistic studies, but they do not entirely reflect the complexity of the *in vivo* environment, where factors such as the immune system and tissue architecture play significant roles. To confirm the protective effects of Klotho against β-cell senescence and fully understand its potential as a therapeutic target, it is essential to validate these findings in more physiologically relevant models. Future studies with primary β-cells or animal models are essential to evaluate Klotho’s potential effects *in vivo*. Additionally, investigating Klotho’s role in different stages of β-cell aging and its interactions with other signaling pathways could offer further insights into its mechanisms. In summary, this is the first study reported KL playing a protective role in β-cell senescence. This might open a new therapeutic opportunity for the prevention of β-cell failure in aging and diabetes.

## Data Availability

The raw data supporting the conclusions of this article will be made available by the authors, without undue reservation.

## Supplementary material

SUPPLEMENTARY FIGURE 1 Viability of untreated and doxorubicin-treated INS-1 cells. Viability was accessed by CCK8 assay. **P* < 0.05.

SUPPLEMENTARY FIGURE 2 The expression of Klotho and IGF-1R in pancreatic β-cells of young and aged mice. Scale bar: 50 μM.

## Abbreviations

T2D, type 2 diabetes; KL, klotho; ROS, reactive oxygen species; ATP, adenosine 5'-triphosphate; IGF-1R, insulin-like growth factor 1 receptor; ECM, extracellular matrix; TNF-α, tumor necrosis factor-α; IL-6, interleukin- 6; IL-1β, interleukin-1 beta; GLUT, glucose transporter type; FBS, fetal bovine serum; SASP, senescence-associated secretory phenotype; GSIS, glucose-stimulated insulin secretion; Dox, doxorubicin; NOD, Non Obese Diabetes; CXCL1 C-X-C, motif chemokine ligand 1; IF, immunofluorescence.
