# Supplementary figures and images for: Klotho protects INS-1 pancreatic β-cells from senescence and enhances mitochondrial function
Source: Front Aging. 2025 Feb 13;6:1512322. doi: 10.3389/fragi.2025.1512322 (PMC11865844; doi:10.3389/fragi.2025.1512322)

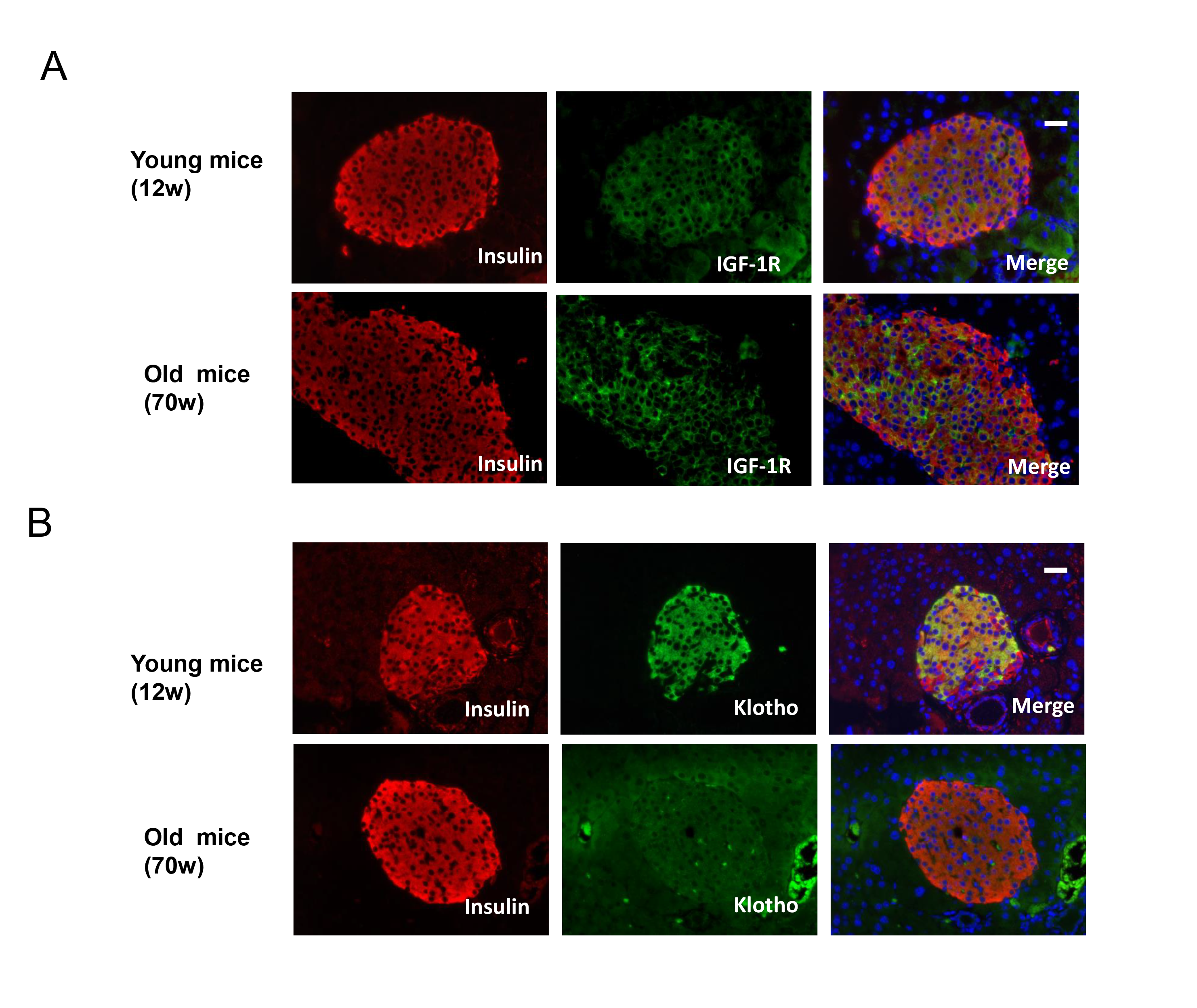

Supplement: Supplementary file 1 [file Image2.tif]

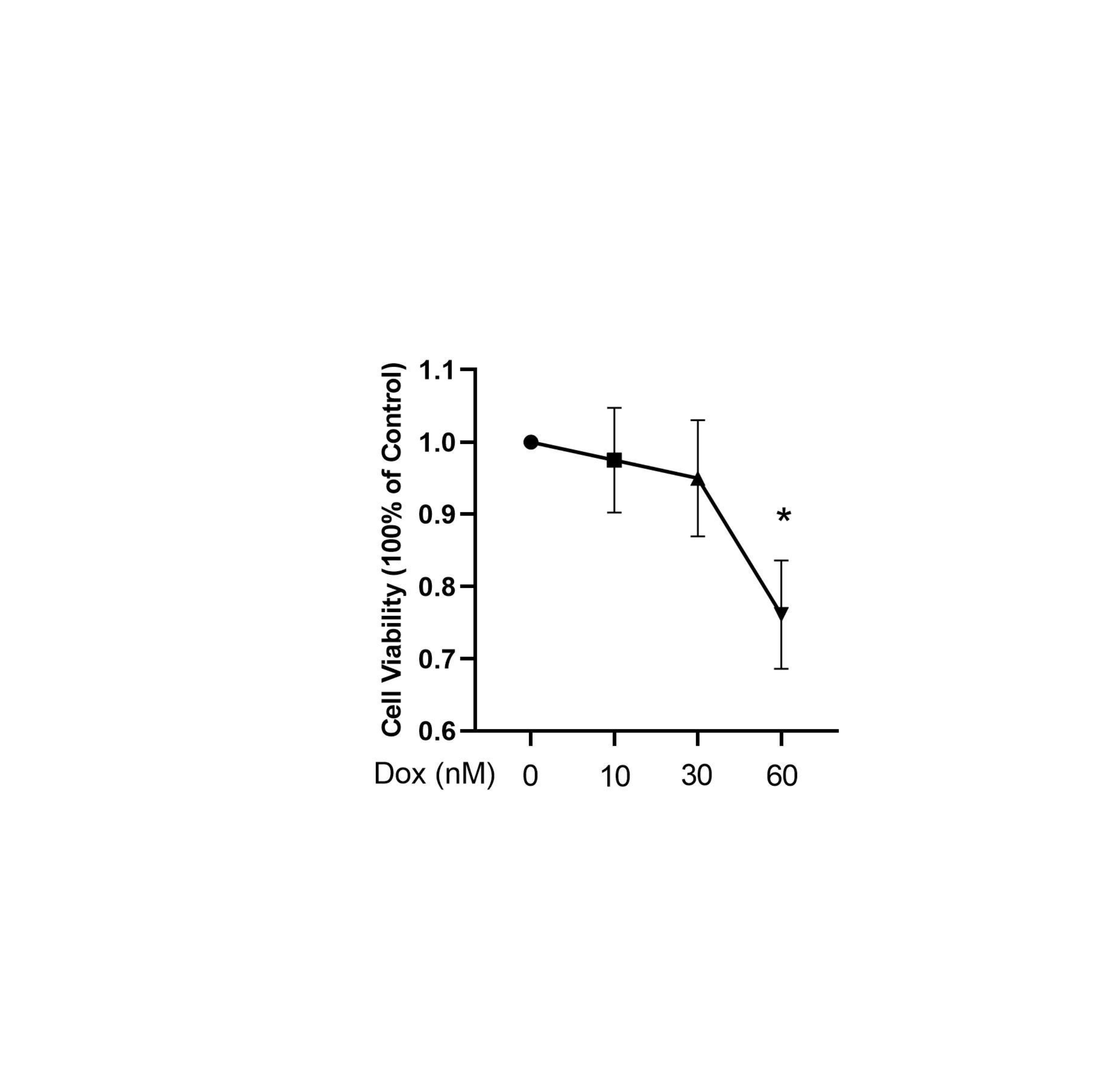

Supplement: Supplementary file 2 [file Image1.tif]
